# The phosphate transporter *PHT4;1* is a salicylic acid regulator likely controlled by the circadian clock protein CCA1

**DOI:** 10.3389/fpls.2014.00701

**Published:** 2014-12-16

**Authors:** Guoying Wang, Chong Zhang, Stephanie Battle, Hua Lu

**Affiliations:** Department of Biological Sciences, University of Maryland Baltimore CountyBaltimore, MD, USA

**Keywords:** circadian clock, defense signaling, programmed cell death, disease resistance, phosphate transporter

## Abstract

The small phenolic compound salicylic acid (SA) plays a critical role in plant defense against broad-spectrum of pathogens. The phosphate transporter gene *PHT4;1* was previously shown to affect SA-mediated defense and its expression is regulated by the circadian clock. To further understand how *PHT4;1* affects SA accumulation, here we analyzed the genetic interactions between the gain-of-function mutant *pht4;1-1* and several known SA mutants, including *sid2-1*, *ald1-1*, *eds5-3*, and *pad4-1*. The genetic analysis was conducted in the *acd6-1* background since the change of *acd6-1* dwarfism can be used as a convenient readout for the change of defense levels caused by impairments in some SA genes. We found that compared with the corresponding double mutants, the triple mutants *acd6-1pht4;1-1ald1-1*, *acd6-1pht4;1-1eds5-3*, and *acd6-1pht4;1-1pad4-1* accumulated lower levels of SA and *PR1* transcripts, suggesting that *PHT4;1* contributes to *acd6-1-*conferred defense phenotypes independently of these known SA regulators. Although some triple mutants had wild type (wt)-like levels of SA and *PR1* transcripts, these plants were smaller than wt and displayed minor cell death, suggesting that additional regulatory pathways contribute to *acd6-1-*conferred dwarfism and cell death. Our data further showed that circadian expression of *PHT4;1* was dependent on CIRCADIAN CLOCK ASSOCIATED 1 (CCA1), a central oscillator component of *Arabidopsis* circadian clock. Recombinant CCA1 protein was demonstrated to bind to the *PHT4;1* promoter in electrophoretic mobility shift assays, suggesting a direct transcriptional regulation of *PHT4;1* by CCA1. Together these results indicate that PHT4;1 is a SA regulator acting independently of several known SA genes and they also implicate a role of the circadian clock mediated by CCA1 in regulating phosphate transport and/or innate immunity in *Arabidopsis*.

## INTRODUCTION

Successful defense against pathogen attacks is critical to plant growth and development. In addition to pre-formed physical and chemical barriers, plants can monitor the presence of pathogens and subsequently activate defense responses to restrict further proliferation and spreading of pathogens. However, it remains challenging to identify genes that control plant defense, understand their mechanisms of action, and determine how they interact to form complex defense networks to orchestrate resistance to invaders.

The small phenolic compound salicylic acid (SA) plays a central role in plant defense signaling (Hammond-Kosack and Jones, 1996; Ryals et al., 1996; Tsuda et al., 2008). Genes that positively regulate SA-mediated defense have been identified in *Arabidopsis*. These genes can be grouped into three types based on their potential biochemical and molecular functions (Lu, 2009; Lu et al., 2009a). The type I SA genes encode enzymes directly involved in SA biosynthesis, which is proposed to take place in the chloroplast and cytoplasm of a cell, involving multiple pathways (Chen et al., 2009; Dempsey et al., 2011). The type I SA gene *ISOCHORISMATE SYNTHASE 1* (*ICS1*), also called SA *Induction-Deficient 2* (*SID2*) and *ENHANCED DISEASE SUSCEPTIBILITY 16* (*EDS16*), contributes to the bulk SA biosynthesis (Nawrath and Metraux, 1999; Wildermuth et al., 2001; Ng et al., 2011). ICS1/SID2/EDS16 protein was shown to be chloroplast-localized (Strawn et al., 2007), indicating that the major SA biosynthetic pathway likely occurs in the chloroplast. To support this notion, the bacterial gene *nahG* that encodes SA hydroxylase to convert SA to the breakdown product catechol (Friedrich et al., 1995), when expressed in the chloroplast, abolishes SA accumulation in the transgenic plants challenged with pathogens or UV light (Fragniere et al., 2011).

Protein products of type II SA genes may not be directly involved in SA biosynthesis. But like SA biosynthetic enzymes (type I), they can influence SA accumulation, possibly through indirect ways, for instance, chemically modifying SA precursors, affecting availability of SA precursors and/or products, influencing expression of type I SA genes, and/or changing activities of SA biosynthetic enzymes. One example of the type II SA regulators is SID1/EDS5, which was localized to the chloroplast membrane and was proposed to transport SA from the chloroplast to the cytoplasm in a cell (Nawrath et al., 2002; Serrano et al., 2013; Yamasaki et al., 2013). The lack of such a SA-transport activity in the *eds5* mutants likely leads to SA accumulation in the chloroplast that feedback-inhibits SA biosynthesis under defense conditions. Indeed, like *sid2* mutants, *eds5* mutants accumulate much reduced SA levels under defense conditions (Nawrath et al., 2002; Ng et al., 2011). Thus these observations further support the idea that the chloroplast is the major site for SA biosynthesis. Additional examples of type II SA regulators include *Accelerated Cell Death (ACD6), AGD2-LIKE DEFENSE 1 (ALD1), EDS1,* and *PHYTOALEXIN DEFICIENT 4* (*PAD4*) (Falk et al., 1999; Jirage et al., 1999; Lu et al., 2003; Song et al., 2004). Loss of function mutations in these SA regulators often lead to EDS and partially reduced SA accumulation upon pathogen insults, compared to wild type (wt) plants. However, the mechanisms by which many of the type II SA regulators act have not been well understood.

Activation of SA signaling often leads to enhanced disease resistance in plants. The type III SA genes act downstream of SA, including SA receptors and signaling transducers. *Non-expressor of PR GENES 1* (*NPR1*) is an example of type III SA genes that has been elegantly studied for its mechanism of action (Dong, 2004; Fu and Dong, 2013). The NPR1 protein has been shown as a key component for SA signaling, overexpression of which confers enhanced disease resistance to a range of pathogens in *Arabidopsis* and some crop plants (Chern et al., 2001; Ekengren et al., 2003; Fitzgerald et al., 2004; Lin et al., 2004; Makandar et al., 2006; Malnoy et al., 2007; Yuan et al., 2007; Quilis et al., 2008; Sandhu et al., 2009; Zhang et al., 2010). Two close homologs of NPR1, NPR3 and NPR4 were recently shown to be SA receptors with different binding affinities to SA (Fu et al., 2012; Fu and Dong, 2013). However, whether or not NPR1 itself is also an SA receptor remains controversial (Wu et al., 2012; Yan and Dong, 2014).

Recent studies showed that two members of a phosphate transporter family, the PHT4 family, were involved in SA regulation. The PHT4 family has six members, five of which (PHT4;1-4;5) are plastid-localized, and one (PHT4;6) is Golgi-localized (Roth et al., 2004; Guo et al., 2008a; Pavon et al., 2008; Cubero et al., 2009). Recombinant proteins of PHT4 family members were demonstrated to have phosphate transport activities (Guo et al., 2008a; Pavon et al., 2008; Cubero et al., 2009). However, only a loss of function mutation in the *PHT4;6* gene but not in other five genes confers enhanced disease resistance to *Pseudomonas syringae* infection and high levels of SA besides reduced salt tolerance (Cubero et al., 2009; Hassler et al., 2012). These results suggest that *PHT4;6* is a negative regulator of SA-mediated defense and is also involved in salt stress response.

The lack of defense and salt tolerance phenotypes in loss of function mutants of other five *PHT4* members is possibly due to functional redundancy among these members. To further support roles of the *PHT4* family members in defense control, we identified a gain of function mutant of the *PHT4;1* gene, *pht4;1-1*, in a genetic screen for *acd6-1* suppressors with a goal to uncover new defense genes (Wang et al., 2011b). *ACD6* encodes an ankyrin repeat protein with transmembrane domain and has been shown as a major determinant of fitness in *Arabidopsis* ecotypes (Lu et al., 2003; Todesco et al., 2010, 2014). *acd6-1* is a small gain-of-function mutant that displays extreme dwarfism, constitutive defense, and spontaneous cell death phenotypes (Rate et al., 1999; Lu et al., 2003). The small size of *acd6-1* is largely in an inverse correlation with the defense level of the plant. This characteristics of *acd6-1* has proven useful in genetic screens to identify novel genes critical for plant defense (Lu et al., 2009a) and in genetic analyses to interrogate interactions between known defense genes (Song et al., 2004; Ng et al., 2011; Wang et al., 2011a). The *pht4;1-1* mutation suppressed high SA accumulation in *acd6-1* and conferred EDS to *P. syringae* infection in the absence of *acd6-1*, which could be rescued by exogenous SA treatment (Wang et al., 2011b). This mutation was caused by a T-DNA insertion that resulted in expression of truncated *PHT4;1* transcripts. Since increasing *PHT4;1* expression by introducing extra copies of *PHT4;1* transgene into wt also conferred EDS (Wang et al., 2011b), we conclude that *pht4;1-1* is a gain of function allele and both PHT4;1 and PHT4;1-1 proteins act similarly as negative regulators of *Arabidopsis* defense. Genetic analysis further indicated that *pht4;1-1* possibly contributed to both SID2-dependent and – independent pathways in regulating *acd6-1-*conferred dwarfism and cell death phenotypes. In addition, *PHT4;1* expression was shown to be regulated by the circadian clock (Guo et al., 2008a; Wang et al., 2011b). Thus we propose that *PHT4;1* is a type II SA regulator, the function of which implicates the circadian clock.

In this report, we further investigated the role of *PHT4;1* in SA regulation and the mechanism of circadian regulation of *PHT4;1*. We examined genetic interactions between *pht4;1-1* and several type II mutants, *ald1-1, eds5-3*, and *pad4-1*, besides the type I SA mutant *sid2-1*. The genetic analysis was done in the *acd6-1* background because the change of *acd6-1* size can be used as a convenient visual readout of functional interactions between the mutants. Our results show that *pht4;1-1* acts additively with *sid2-1*, *ald1-1*, *eds5-3*, and *pad4-1* to regulate *acd6-1* dwarfism, cell death, and/or defense responses, suggesting that *PHT4;1* has distinct function from these other SA regulators. To elucidate the mechanism by which *PHT4;1* is circadian clock-regulated, we tested the hypothesis that *PHT4;1* is a direct target of the core component of *Arabidopsis* circadian clock CIRCADIAN CLOCK ASSOCIATED 1 (CCA1). Our data support the hypothesis and underscore a possible role of the circadian clock mediated by CCA1 in regulating the function of *PHT4;1* in phosphate transport and/or innate immunity control in *Arabidopsis*.

## MATERIALS AND METHODS

### PLANT MATERIALS

All *Arabidopsis* plants used in this report are in Columbia-0 background. Plants were grown in growth chambers with a 12 h light/12 h dark cycle, light intensity at 200 μmol m^-2^ s^-1^, 60% humidity, and 22°C. The triple mutant *acd6-1pht4;1-1sid2-1* was previously described (Wang et al., 2011b). Additional triple mutants were made by crossing *acd6-1pht4;1-1* with *acd6-1ald1-1*, *acd6-1eds5-3,* or *acd6-1pad4-1* and selected for homozygotes by polymerase chain reaction (PCR) with appropriate primers (Ng et al., 2011).

### RNA ANALYSIS

Whole plants of each genotype at 25-day old were harvested at ZT1 (1 h after lights on) for RNA extraction. For circadian clock-regulated gene expression, plants grown in 12 h L/12 h D were transferred to constant light (LL) and harvested starting at ZT1 at a 4 h interval for 48 h. RNA extraction and northern blotting were performed as described (Ng et al., 2011). Radioactive probes were made by PCR with antisense primers specific to individual gene fragments in the presence of [^32^P] dCTP. Primers used for making the *PR1* probe are PR1_sense 5′ GTAGGTGCTCTTGTTCTTCCC 3′ and PR1_antisense 5′ CACATAATTCCCACGAGGATC 3′ and for making the *PHT4;1* probe are PHT4;1_sense 5′ ATGAACGCGAGAGCTCTTCTTTGCTC 3′ and PHT4;1_antisense 5′ AATCGATTATCTTCTCTCCGGTTG 3′.

### SA MEASUREMENT

SA was extracted from 25-day old plants and quantified by a high-performance liquid chromatography (HPLC) instrument as previously described (Ng et al., 2011; Wang et al., 2011a).

### CELL DEATH STAINING

The sixth or seventh leaves of 25-day old plants were harvested for trypan blue staining as described (Ng et al., 2011). Stained leaves were washed with 50% ethanol and mounted on glass slides with cover slips for photographing with a complementary metal–oxide–semiconductor (CMOS) camera connected to a dissecting microscope (Leica M205 FA, Leica Microsystems, Germany).

### PURIFICATION OF CCA1-GST RECOMBINANT PROTEIN FROM *Escherichia coli*

The pGEX-CCA1 construct containing CCA1-GST in the pGEX-3X vector was a kind gift from Steve Kay at University of South California. pGEX-CCA1 was transformed into the *Escherichia coli* strain BL21(DE3)-pLysS to express the recombinant protein. A single colony was picked for overnight culture in 5 ml LB media, which was subsequently added into 500 ml LB media for further culture. At OD_600_ = 0.5, the culture was treated with 0.4 mM isopropyl β-D-1-thiogalactopyranoside for 3 h followed by harvesting by centrifugation at 8000 g for 10 min at 4°C. The pellet was resuspended in 25 ml ice–cold 1 X PBS containing 1% Triton X-100 and 2x protease inhibitor (Roche, LOT# 14549800) and lysed by sonication on ice. The sonication condition was 30 s on followed by 30 s off at 30% amplitude for 20 cycles, using Virsonic Cell Disruptor (Model 16-850, The Virtis Co., New York). Cell lysates were collected by centrifugation at 8000 g for 10 min at 4°C. The supernatant was loaded onto a 2 ml glutathione spin column (Pierce, Product # 16107), incubated at 4°C for 30 min on a rocking platform. The column was washed with 10x bed volumes of equilibration/wash buffer (125 mM Tris, 150 mM NaCl, pH 8.0). The CCA1-GST recombinant proteins were eluted with elution buffer (10 mM glutathione, 125 mM Tris, 150 mM NaCl, pH 8.0), according to manufacturer’s instruction (Pierce, Product # 16107). Purified CCA1-GST protein was verified on a 6% SDS-PAGE gel and aliquoted into 30 μl per microcentrifuge tube for storage at -80°C.

### ELECTROPHORETIC MOBILITY SHIFT ASSAYS

Three DNA fragments (probes) from the *PHT4;1* promoter were generated by PCR amplification, purified, and used for CCA1-GST binding assays. Probe 1 (396 bp) covers from -348 to +48 bp relative to the ATG start site of the *PHT4;1* promoter (primers 5′ TTGTTATTGGTATTGCCGTATTATTGTA 3′, and 5′ GTAGAGAGAGTGAATATTTGAAGA 3′). Probe 2 (118 bp) covers from -348 to -230 bp relative to the ATG start site of the *PHT4;1* promoter (primers 5′ TTGTTATTGGTATTGCCGTATTATTGTA 3′, and 5′ GTTAGCTTACGAGCATAAATTGC 3′). Probe 3 (117 bp) covers from -69 to +48 bp relative to the ATG start site of the *PHT4;1* promoter (primers 5′ AATCAATTCCTCTCTCTTAAAACAAA 3′, and 5′ GTAGAGAGAGTGAATATTTGAAGA 3′). The negative probe *PHT4;1-NC* (without CCA1 binding site) was generated by PCR amplification of the region from +134 to +668 of the *PHT4;1* gene (primers 5′ CTACCCGCGAAATAGGTCCAGTG 3′, and 5′ ATCAACAAACCACTGATTCAACTACACTT 3′). Probes (60 ng each) were end-labeled with γ-[^32^P]-dATP, using T4 polynucleotide kinase (Thermo Scientific, product # EK0031) in the following reaction: 2 pmol DNA fragment, 2 μl 10x forward reaction buffer, 4 pmol γ-[^32^P]-dATP, 1 μl T4 PNK, in a total volume of 20 μl. The reaction was carried out at 37°C for 30 min, then added 1 μl of 0.5 M EDTA (pH 8.0) and incubated at 75°C for 10 min to terminate the reaction. Labeled DNA probes were purified by using a PCR purification kit (Qiagen, cat#28104) and eluted with 30 μl sterile water. Binding reactions were carried out as following: 2 μl 5X electrophoretic mobility shift assays (EMSA) buffer [125 mM HEPES-KOH (pH 7.5), 12.5 mM DTT, 5 mM PMSF, 250 mM KCl], 2 μl 50% glycerol, 1 μl 1 μg/ul poly-dIdC, 30–90 ng CCA1-GST recombinant protein, 1 μl labeled probe, in a total volume of 10 μl. For a competition assay, excessive amount of a corresponding cold probe or the negative probe *PHT4;1-NC* at the indicated concentrations was added to a binding reaction. Both binding and competition reactions were incubated on ice for 20 min before being immediately loaded onto a 6% non-denaturing polyacrylamide gel, prepared in 0.5X TBE buffer [40 mM Tris-Cl (pH 8.3), 45 mM boric acid, 1 mM EDTA]. Electrophoresis was conducted at 100 V for ∼1 h at room temperature to separate free probes from DNA-protein complexes. The gels were dried on a gel dryer (Hoefer, model SE1160) at 80°C for 1 h followed by exposure to X-ray film for 2–4 days.

## RESULTS

### *PHT4;1* INTERACTS ADDITIVELY WITH MULTIPLE SA REGULATORS TO AFFECT *ACD6-1* DWARFISM

**FIGURE 1:**
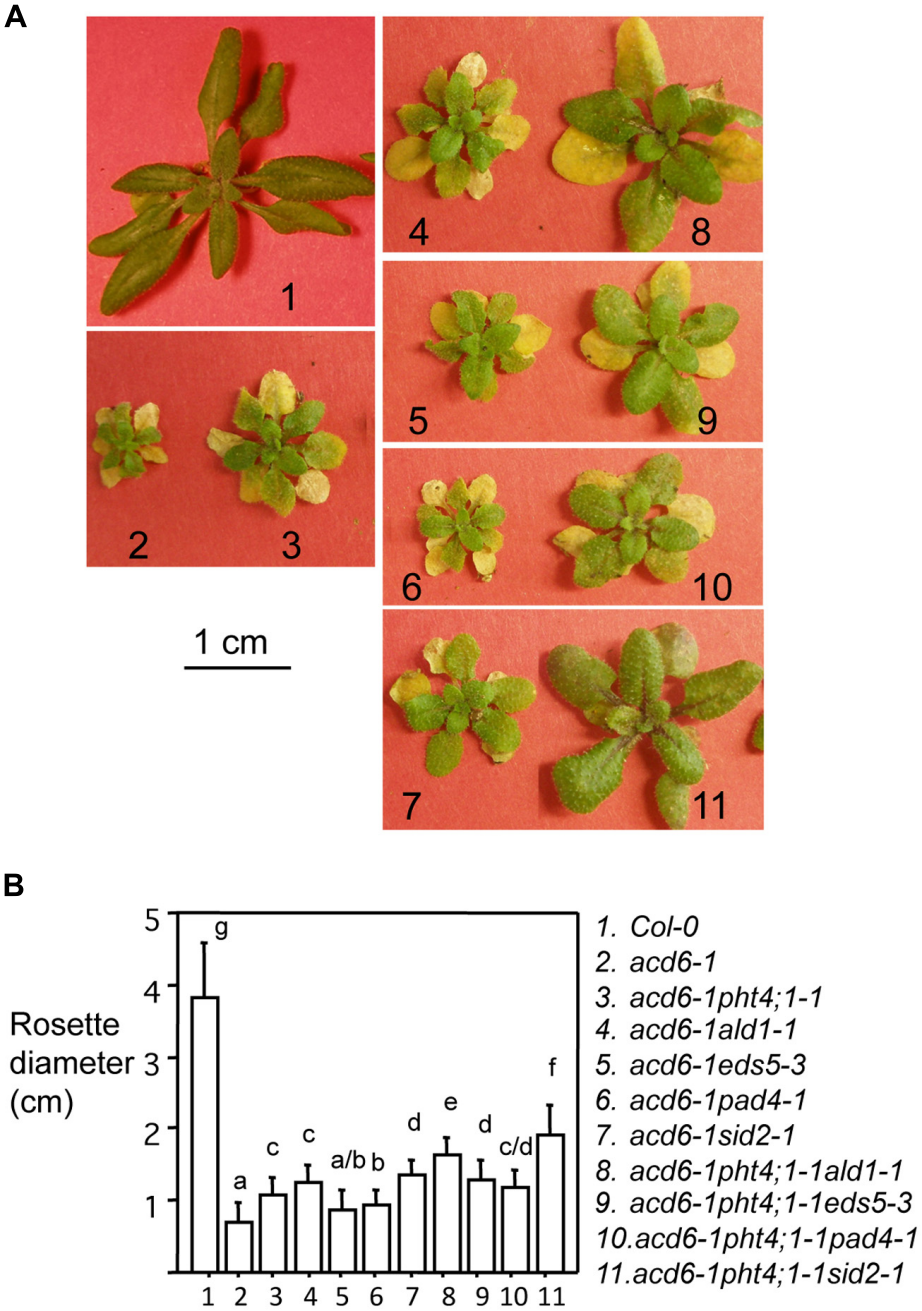
***pht4;1-1* acts additively with SA mutants to suppress *acd6-1* dwarfism**. **(A)** Pictures of 25-day old plants. The single mutants *pht4;1-1, ald1-1, eds5-3*, *pad4-1*, and *sid2-1* are morphologically similar to Col-0 (not shown). The scale bar represents 1 cm and applies to all panels. **(B)** Plant size measurement. Plants shown in **(A)** were measured for their rosette diameters. Statistical analysis was performed with Student’s *t*-test (StatView 5.0.1). Different letters indicate significant difference among the samples (*P* < 0.05; *n* = 10).

Our previous data suggest that the *PHT4;1* gene acts upstream of SA to regulate SA accumulation (Wang et al., 2011b). To further investigate the role of *PHT4;1* in SA regulation, we sought to examine genetic interactions between the gain of function mutant *pht4;1-1* and mutants disrupting type II SA genes, *ADL1*, *EDS5*, and *PAD4* (Jirage et al., 1999; Nawrath et al., 2002; Song et al., 2004). We crossed *pht4;1-1* to these mutants in the *acd6-1* background because the small size of *acd6-1* is sensitized to the change of defense levels and thus *acd6-1* can be conveniently used to dissect the functional relationship among SA genes (Song et al., 2004; Ng et al., 2011). A previous similar experiment showed that *pht4;1-1* acts additively with the type I SA mutant *sid2-1* in affecting *acd6-1* dwarfism (Wang et al., 2011b). We found here that similar to *acd6-1pht4;1-1sid2-1*, the triple mutants *acd6-1pht4;1-1ald1-1*, *acd6-1pht4;1-1eds5-3*, and *acd6-1pht4;1-1pad4-1* were significantly larger than their corresponding double mutants (**Figures 1A,B**), suggesting that *PHT4;1* acts additively with multiple SA regulators in influencing *acd6-1* dwarfism.

### *PHT4;1* INTERACTS ADDITIVELY WITH MULTIPLE SA REGULATORS TO AFFECT DEFENSE PHENOTYPES AND CELL DEATH IN *ACD6-1*

**FIGURE 2:**
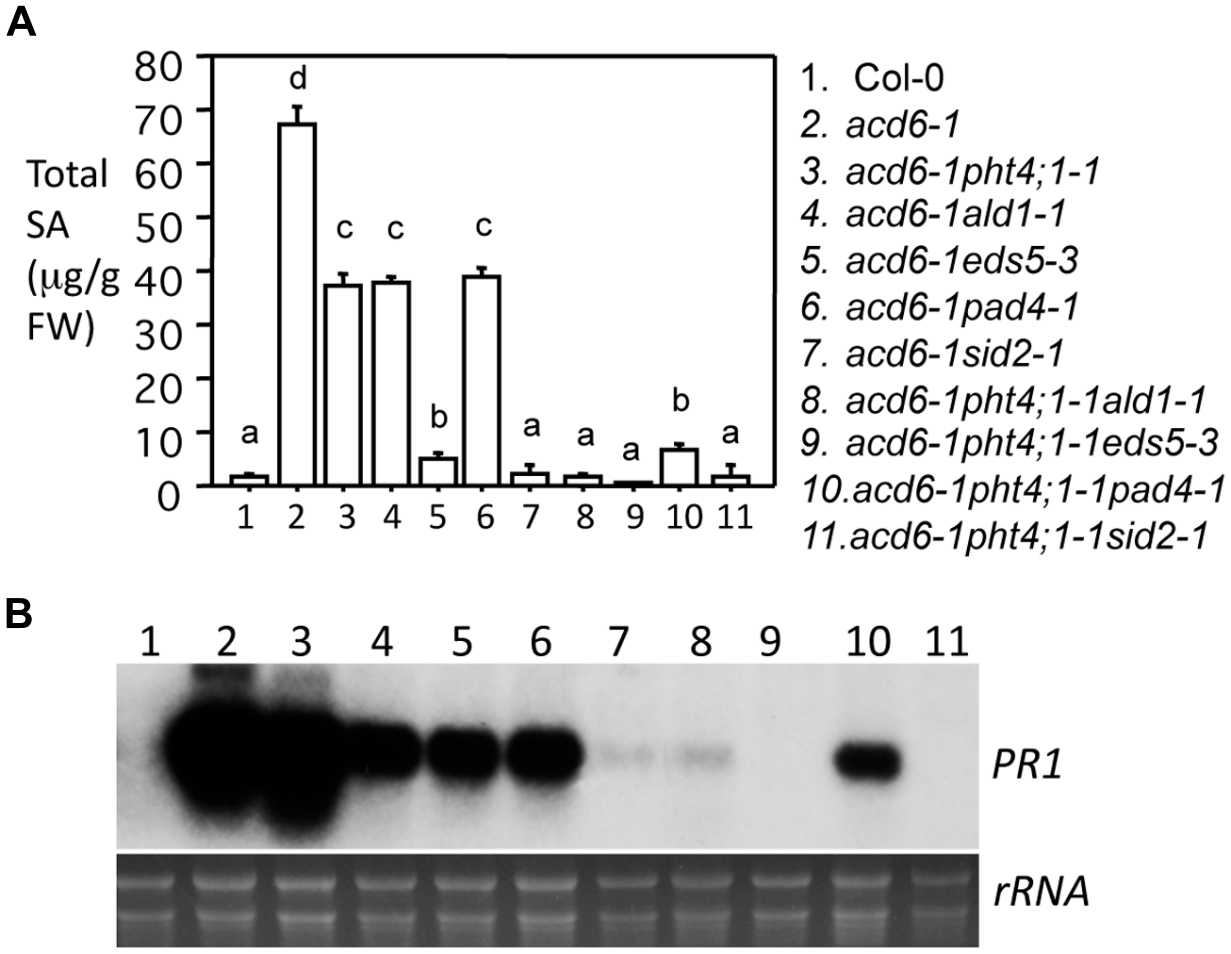
***pht4;1-1* acts additively with SA mutants to suppress SA accumulation and *PR1* expression in *acd6-1*.** Twenty five-day old plants were harvested for SA extraction followed by HPLC analysis and RNA preparation followed by northern blotting. **(A)** SA quantitation. Statistical analysis was performed with Student’s *t*-test (StatView 5.0.1). Different letters indicate significant difference among the samples (*P* < 0.05; *n* = 3). **(B)** *PR1* expression. rRNA was shown as a loading control.

Since previous studies showed that the dwarfism of *acd6-1* is grossly in reverse correlation with the defense level of the plant (Song et al., 2004; Lu et al., 2009a; Ng et al., 2011; Wang et al., 2011a), the increased size of the triple mutants shown in **Figure 1** suggests reduced defense of the plants. To further test this, we measured SA levels and expression of the defense marker gene *PR1* in these plants. Indeed we found that *acd6-1pht4;1-1ald1-1* and *acd6-1pht4;1-1eds5-3* accumulated near wt-level of SA and *PR1* transcripts (**Figures 2A,B**). *acd6-1pht4;1-1pad4-1,* on the other hand, had much reduced SA level than the two parental double mutants but this level was still significantly higher than that seen in wt. Expression of *PR1* was only slightly reduced in *acd6-1pht4;1-1pad4-1*, compared with the corresponding double mutants (**Figure 2B**). These results indicate that *PHT4;1* has distinct function from these type II SA genes in regulating SA accumulation and *PR1* expression. Consistent with a major role of *SID2* in SA biosynthesis (Wildermuth et al., 2001; Ng et al., 2011), we found that SA accumulation and *PR1* expression in *acd6-1pht4;1-1sid2-1* were comparable to those of *acd6-1sid2-1* and wt. We also noticed that the near-wt level of SA in some triple mutants (*acd6-1pht4;1-1ald1-1, acd6-1pht4;1-1eds5-3*, and *acd6-1pht4;1-1sid2-1*) was not correlated with a complete suppression of *acd6-1* dwarfism, suggesting there are additional pathways independent of SA contributing to plant size regulation in *acd6-1*.

**FIGURE 3:**
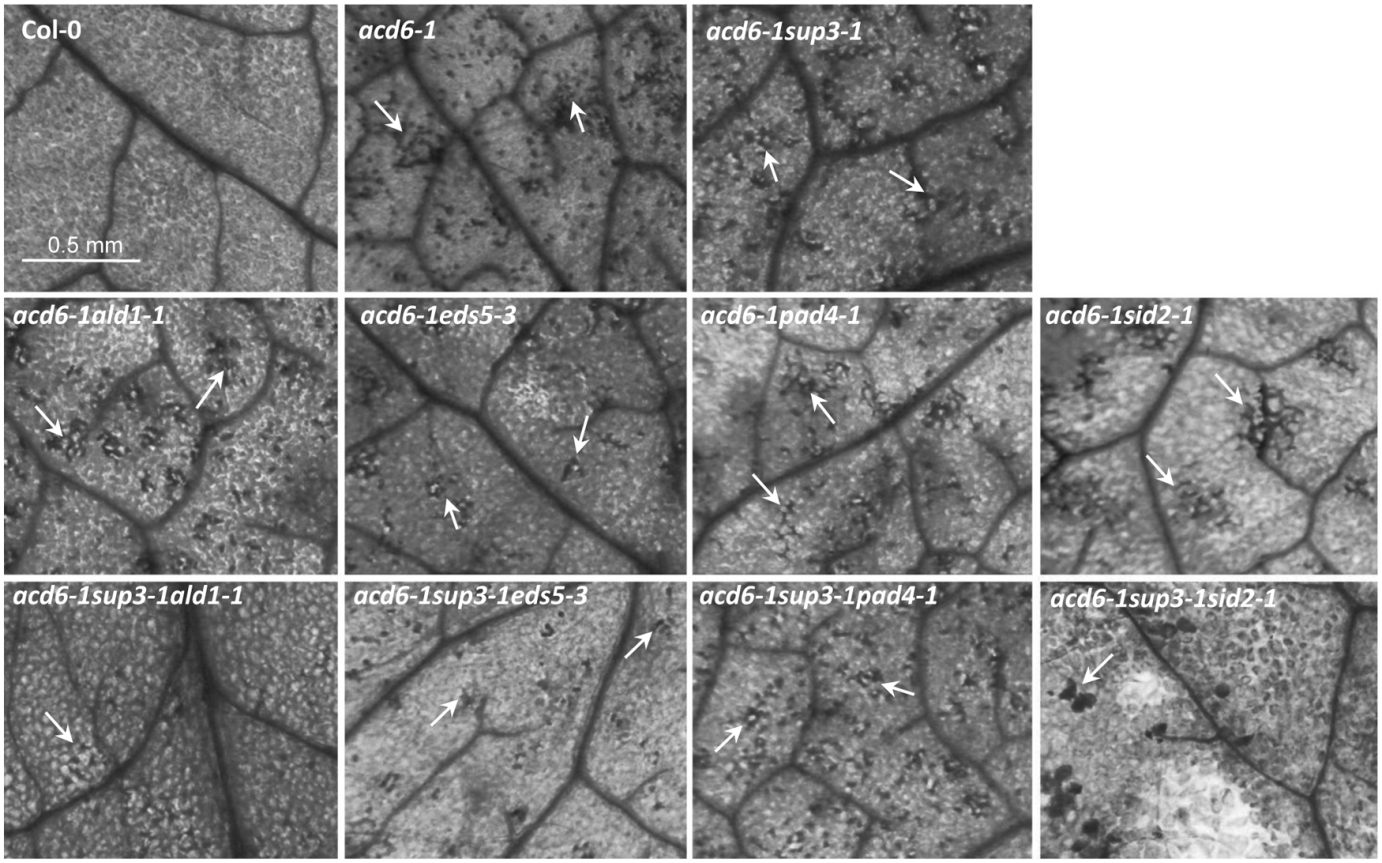
***pht4;1-1* acts additively with SA mutants to suppress cell death in *acd6-1*.** The sixth or seventh leaves of 25-day old plants were harvested for trypan blue staining as described (Ng et al., 2011). Stained leaves were photographed with a CMOS camera connected to a dissecting microscope (Leica M205 FA, Leica Microsystems, Germany). Cell death is shown in the dark stained spots or patches on a leaf (arrows). At least four leaves of each genotype were stained and examined for cell death. No cell death was detected in *ald1-1*, *eds5-3*, *pad4-1, sid2-1*, and *pht4;1-1* (data not shown). The scale bar represents 0.5 mm and applies to all panels.

Besides dwarfism and enhanced defense phenotypes, the *acd6-1* mutant displays severe cell death, even in the absence of pathogen challenge. Suppression of *acd6-1-*conferred dwarfism and defense phenotypes is usually associated with reduced cell death (Song et al., 2004; Lu et al., 2009a; Ng et al., 2011; Wang et al., 2011a). Consistent with this previous observation, we found that triple mutants *acd6-1pht4;1-1ald1-1, acd6-1pht4;1-1eds5-3*, and *acd6-1pht4;1-1sid2-1* had substantially reduced but not abolished cell death on their leaves when the plants were stained with trypan blue to visualize cell death (**Figure 3**). Since these mutants accumulated wt-level SA, like plant-size regulation, cell death formation in these plants could be influenced by additional SA-independent pathway(s). Interestingly *acd6-1pht4;1-1pad4-1* displayed similar cell death as *acd6-1pht4;1-1*. This could be due to the relatively high level of SA presented in the triple mutant. Alternatively *PHT4;1* and *PAD4* could act in the same pathway to affect cell death of *acd6-1*.

### CIRCADIAN EXPRESSION OF *PHT4;1* IS CCA1-DEPENDENT

**FIGURE 4:**
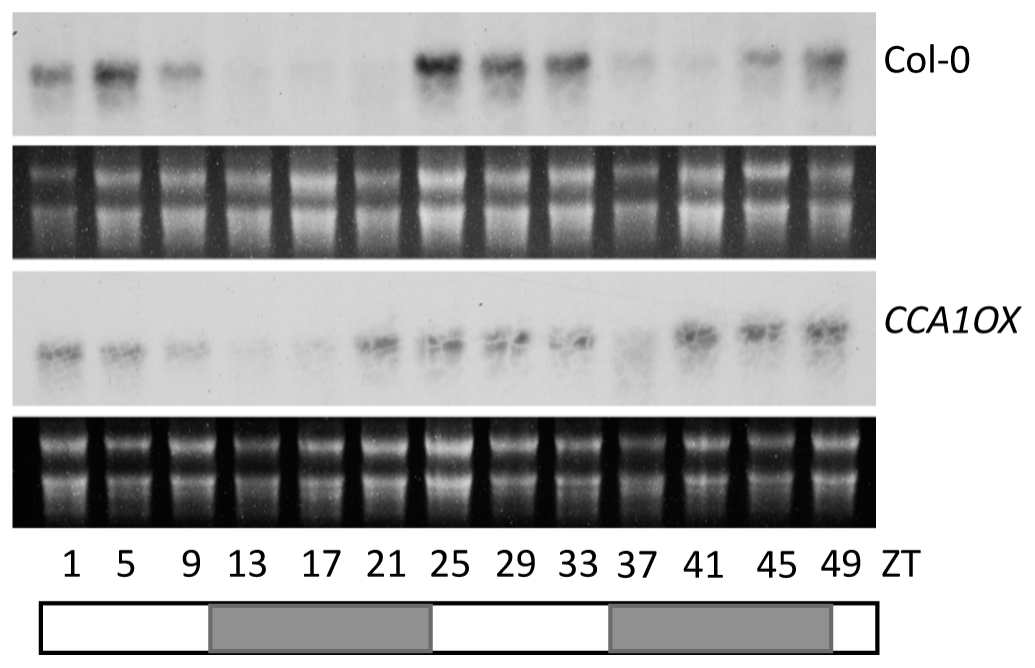
**Circadian expression of *PHT4;1* is CCA1-dependent.** Twenty five-day-old Col-0 and *CCA1ox* plants grown in a chamber with a 12 h light/12 h dark cycle and 22°C were transferred to LL at 22°C. Starting at ZT1, plants were harvested at every 4 h for 48 h for RNA extraction followed by northern blotting. White boxes indicate subjective light periods and gray boxes indicate subjective dark periods in LL. rRNA was shown as a loading control.

Expression of *PHT4;1* was previously shown to be regulated by the circadian clock (Guo et al., 2008a; Wang et al., 2011b). Such a circadian expression pattern persisted in *pht4;1-1* and *acd6-1* mutants and in the presence of *P. syringae* challenge (Wang et al., 2011b). Consistent with being regulated by the circadian clock, the *PHT4;1* promoter has two *cis*-elements (the CBS motifs), starting at -17 and -281 bp positions, respectively, that are putative binding sites for the core clock component CCA1 (Alabadi et al., 2002; Green and Tobin, 2002; Michael and McClung, 2002). Thus we hypothesized that CCA1 directly targets *PHT4;1* promoter for expression regulation. To test this hypothesis, we first examined *PHT4;1* expression in *CCA1* overexpressing (*CCA1ox*) plants, which display arrhythmic clock activity in both constant light (LL) and light/dark (LD) conditions (Wang and Tobin, 1998; Zhang et al., 2013). We found that when the plants were transferred from LD to LL, *PHT4;1* demonstrated a circadian expression pattern in wt Col-0. However, *CCA1ox* disrupted this expression pattern of *PHT4;1* (**Figure 4**). This result indicates a role of CCA1 in controlling *PHT4;1* expression but could not pinpoint whether such an effect is direct or indirect.

**FIGURE 5:**
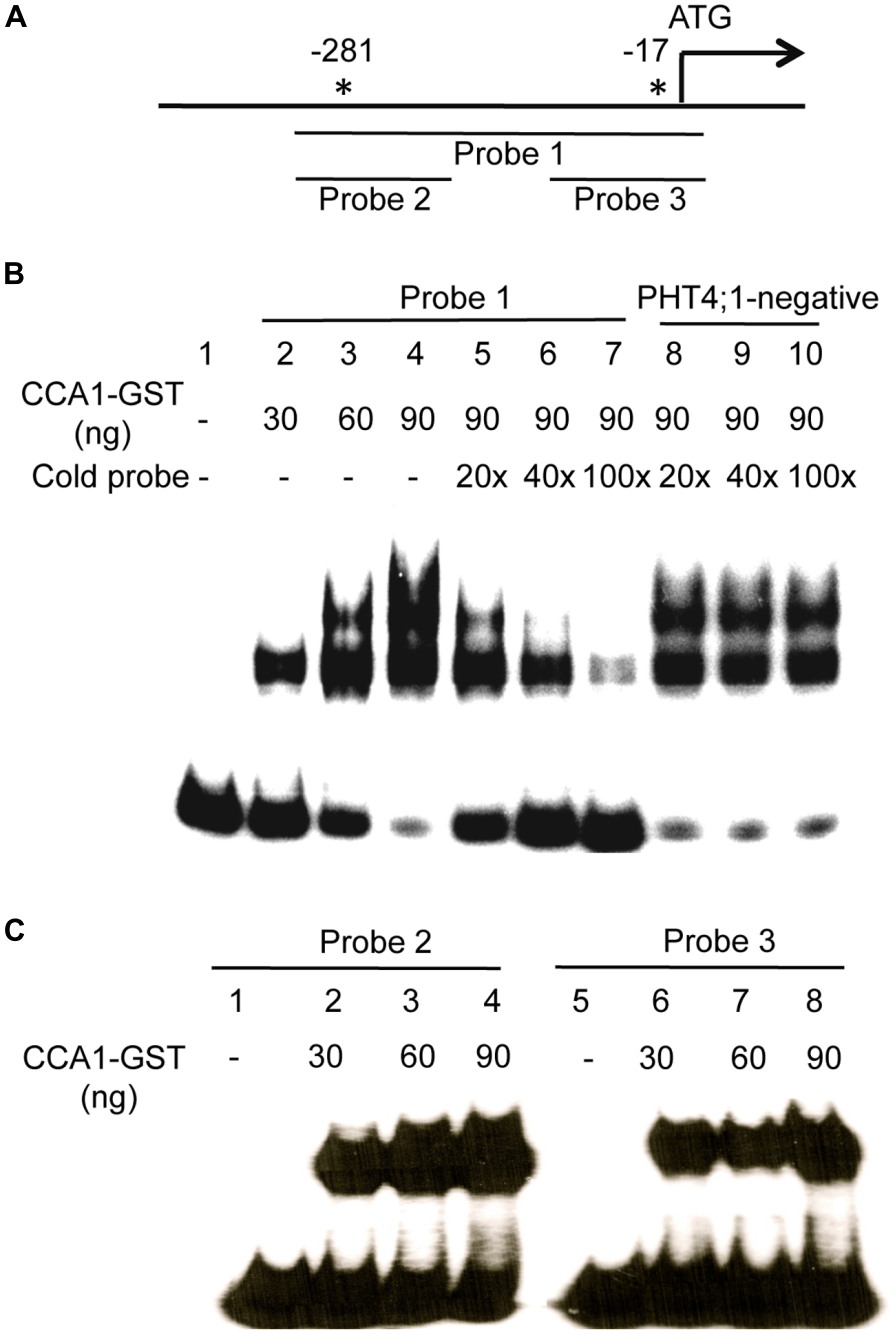
**CCA1 binds to the *PHT4;1* promoter in EMSA. (A)** Positions of the *PHT4;1* promoter fragments (probes) and the CBS motifs (asterisks). Positions are relative to the translation start site (ATG). **(B)** EMSA with probe 1. **(C)** EMSA with probe 2 and probe 3. The probes were end-labeled with γ-^32^P and incubated with purified recombinant CCA1-GST protein. For competition assays in **(B)**, unlabeled fragments (probe 1 or PHT4;1-negative) at the indicated folds more than the input (isotope-labeled probe 1) were added to the binding reactions. The reactions were resolved on 6% native PAGE gels followed by gel drying and exposure to X-ray film.

To further test if CCA1 directly binds to the *PHT4;1* promoter, we conducted EMSA with CCA1-GST recombinant protein and *PHT4;1* promoter fragments. The probe 1 is a *PHT4;1* fragment containing two CBS motifs (**Figure 5A**). We found that probe 1 was bound by recombinant CCA1-GST protein, resulting in slower moving bands containing protein-DNA complexes (**Figure 5B**, lane 2–4). Unlabeled probe 1 could compete with isotope-labeled probe 1 for CCA1-GST binding in a dose-dependent manner (**Figure 5B**, lane 5–7). However, excess amount of a negative fragment (PHT4;1-negative) from *PHT4;1* without a CBS motif did not compete with isotope-labeled probe 1 in CCA1-GST binding (**Figure 5B**, lane 8–10). These results suggest that the binding between probe 1 and CCA1-GST protein is specific. We also noticed that there were two shifted bands in most lanes from probe 1 and CCA1-GST binding reactions (**Figure 5B**, lane 3–5 and 8–10). We speculated that both CBS motifs in probe 1 can be bound by CCA1-GST when the protein is present in abundance. To test this, we incubated two shorter *PHT4;1* promoter fragments (probe 2 and probe 3), containing only one CBS motif each, with CCA1-GST (**Figure 5C**). Indeed, both probe 2 and 3 were bound by CCA1-GST, forming a single DNA-protein complex that separated from the free probes. Thus these *in vitro* binding assays support our hypothesis that *PHT4;1* is a direct target of CCA1.

## DISCUSSION

In this study, we took biochemical, genetic, and molecular approaches to further investigate the function of the phosphate transporter gene *PHT4;1*. Our results show that *PHT4;1* genetically interacts with several SA genes, including *SID2, ALD1*, *EDS5*, and *PAD4*, in regulating defense responses. In addition, we show that circadian expression of *PHT4;1* is dependent on the circadian clock protein CCA1, which could directly bind to the *PHT4;1* promoter. These results corroborate the role of *PHT4;1* in defense regulation and also suggest that the circadian clock gene *CCA1* regulates phosphate transport and/or defense responses, possibly through influencing *PHT4;1*-mediated pathway.

Our previous study indicated that *PHT4;1* is a negative defense regulator acting upstream of SA (Wang et al., 2011b). Genetic analysis conducted here further showed that *pht4;1-1* acts additively with SA mutants *ald1-1*, *eds5-3,* and *pad4-1* to suppress high levels of SA accumulation and *PR1* expression in *acd6-1* (**Figure 2**). Thus *PHT4;1* likely functions in a separate pathway from *ALD1, EDS5,* and *PAD4* in regulating these defense outputs. Consistent with this notion, expression of *ACD6*, *ALD1*, and *PAD4* are inducible by SA treatment, suggesting that these genes are involved in signal amplification loops with SA (Nawrath et al., 2002; Lu et al., 2003; Song et al., 2004). However, expression of *PHT4;1* is not affected by SA treatment (data not shown), suggesting that unlike *ACD6*, *ALD1*, and *PAD4*, *PHT4;1* is not part of SA-signal amplification loop. Together these results further support a previous notion that there are multiple pathways affecting SA-mediated defense in *Arabidopsis* (Song et al., 2004; Ng et al., 2011; Wang et al., 2011a). Interestingly although some triple mutants show wt-like levels of SA and *PR1* expression, none of these triple mutants revert to wt-like phenotypes in terms of plant size and cell death (**Figures 1** and **3**). These results suggest that the regulation of plant size and cell death can be uncoupled from that of some defense phenotypes in *acd6-1*. Additional SA-independent pathway(s) could contribute to the regulation of plant size and cell death formation in *acd6-1*.

While the gain of function mutant *pht4;1-1* displayed compromised defense phenotypes, the loss of function alleles of *PHT4;1* did not show altered defense responses (Wang et al., 2011b). This can be explained by possible functional redundancy among some PHT4 family members. Indeed, PHT4;1 and four other members in the family share high levels of homology and are all plastid-localized. Functional redundancy among these members could prevent manifestation of defense phenotypes in single loss of function mutants. So far only one disrupted member, *PHT4;2*, showed small effects on plant growth (Irigoyen et al., 2011). Besides *pht4;1* loss of function mutants, available single loss of function mutants of *PHT4;4* and *PHT4;5* are indistinguishable from wt in morphology and defense responses (data not shown).

While five plastid-localized PHT4 family members could share redundant function, the sixth member of the family, PHT4;6, might be functionally divergent from other members in the family. PHT4.6 is localized to the Golgi and was shown to have Pi transport activity in the Golgi (Guo et al., 2008a; Cubero et al., 2009). A single loss of function mutation in *PHT4;6* results in enhanced disease resistance to *P. syringae* infection, dwarfism, and reduced salt tolerance (Cubero et al., 2009; Hassler et al., 2012). The *pht4;6* mutant also accumulates modestly higher levels of SA than wt. Thus like PHT4;1, PHT4;6 is also a negative regulator of plant defense.

The involvement of two members of the PHT4 family in defense suggests a possibility that phosphate transport is critical for host-pathogen interactions. Phosphorus (P) is essential for plant growth and development. However, plants do not produce P but take up inorganic phosphate ion (Pi) from the soil to the root, reallocate Pi to different tissue and cell types, and redistribute Pi to different organelles within a cell in order to fulfill the Pi requirement for cellular functions. These processes are mediated by phosphate transporters to maintain phosphate homeostasis and the normal function of cells. At least five phosphate transporter families, PHT1, PHT2, PHT3, PHT4, and pPT, have been reported in *Arabidopsis* (Poirier and Bucher, 2002; Guo et al., 2008b). Among these phosphate transporter families, only mutations in some *PHT1* genes and one *PHT2* gene resulted in alterations in Pi concentration *in planta* (Versaw and Harrison, 2002; Shin et al., 2004; Gonzalez et al., 2005). The *PHT1* genes encode plasma membrane-localized high affinity Pi/H^+^ symporters and are expressed abundantly in the root (Karthikeyan et al., 2002; Mudge et al., 2002). The *PHT2* gene encodes a chloroplast-localized phosphate transporter and is highly expressed in the green tissue (Versaw and Harrison, 2002). Based on these tissue- and cell-specific expression patterns, *PHT1* was proposed to acquire Pi from the root whereas *PHT2* was proposed to influence the reallocation of phosphate within different tissues of a plant. PHT4 and other phosphate transporter families have not been reported to have a major effect on phosphate concentration at the whole plant level. Except two members of the *PHT4* family (*PHT4;1* and *PHT4;6*), none of the other phosphate transporter genes have been demonstrated a role in defense regulation. Therefore it is currently unknown whether perturbation of phosphate concentration *in planta* could result in altered defense responses. However, there is evidence to support a connection between altered phosphate signaling and defense control. One example is the *SIZ1* gene encoding a SUMO E3 ligase that targets PHR1, a MYB transcriptional activator critical for phosphate response. A *siz1* mutant demonstrated reduced phosphate response and enhanced disease resistance (Rubio et al., 2001; Miura et al., 2005; Lee et al., 2007; Jin et al., 2008).

Since PHT4;1 is not known to perturb phosphate concentration at the whole plant level, the defense phenotypes observed in the *pht4;1-1* mutant could be caused by altered PHT4;1 transport activity at the subcellular level. *PHT4;1* is mainly expressed in the shoot tissue (Guo et al., 2008a,b). Pavon et al. (2008) showed that the PHT4;1 protein was localized to the thylakoid member of the chloroplast and thus proposed that PHT4;1 transports Pi across thylakoid lumen and stroma in the chloroplast, using its Na^+^ and/or H^+^- dependent phosphate transporter activity (Guo et al., 2008b). In another study, Roth et al. (2004) localized PHT4;1 to the inner membrane of the plastid. Although the precise localization of PHT4;1 remains to be determined, these studies pointed to the connection of PHT4;1 with the chloroplast, the central organelle for photosynthesis and many secondary and primary metabolisms, including SA biosynthesis. It is conceivable that Pi transported by PHT4;1 could directly or indirectly affect SA biosynthetic pathways or proteins/processes that affect SA accumulation. Such function of PHT4;1 could be shared by other four plastid-localized PHT4 family members (PHT4;2-4;5). However, Golgi-localized PHT4;6 may influence SA accumulation and SA-mediated defense through a different mechanism from that used by PHT4;1.

The observation of circadian clock regulated *PHT4;1* expression has prompted us to elucidate the role of the circadian clock in defense control (Guo et al., 2008a; Wang et al., 2011b; Zhang et al., 2013). The circadian clock is an internal time measuring machinery important for development and fitness of plants (Green et al., 2002; Michael et al., 2003; Dodd et al., 2005; Ni et al., 2009; Graf et al., 2010; Dong et al., 2011). Increasing evidence supports a role of the circadian clock in defense regulation. First, like *PHT4;1*, expression of some defense genes were reported to be under the circadian clock control (Wang et al., 2001, 2011b; Sauerbrunn and Schlaich, 2004; Weyman et al., 2006; Roden and Ingle, 2009). Second, wt *Arabidopsis* shows temporal variations in a day in its susceptibility to *P. syringae* infection and such variations can be disrupted by overexpression of *CCA1* (Bhardwaj et al., 2011). Third, misexpression of several core clock genes, including *CCA1*, its close homolog *LATE ELONGATED HYPOCOTYL* (*LHY*) (Alabadi et al., 2002; Mizoguchi et al., 2002; Lu et al., 2009b), and *TIME FOR COFFEE* (Hall et al., 2003; Ding et al., 2007), leads to compromised resistance to the bacterial pathogen *P. syringae* and/or to the oomycete pathogen *Hyaloperonospora arabidopsidis* (*Hpa*) (Bhardwaj et al., 2011; Wang et al., 2011c; Shin et al., 2012; Zhang et al., 2013). Data from our study further indicate that defense activation can reciprocally regulate clock activity, suggesting crosstalk between the circadian clock and plant innate immunity (Zhang et al., 2013).

Both experimental studies and *in silico* analysis of circadian clock-regulated gene expression indicate that *PHT4;1* is the only member in the *PHT4* family that demonstrates a robust circadian expression pattern (Mockler et al., 2007; Guo et al., 2008a, and data not shown). We presented here evidence to further demonstrate that *PHT4;1* could be a direct transcriptional target of the circadian clock protein CCA1 (**Figures 4** and **5**). Interestingly, while we show here that *PHT4;1* is an SA regulator that acts independently of several known SA genes, our previous study indicated that the clock genes *CCA1* and *LHY* acted in a SA-independent manner in defense regulation (Zhang et al., 2013). The *cca1-1lhy-20* double mutations suppressed *acd6-1-*conferred constitutive defense but not its dwarfism and high SA accumulation. Such a discrepancy in terms of SA regulation by PHT4;1 and CCA1 suggest that CCA1-regulation of *PHT4;1* might be important for phosphate transport activity of PHT4;1 but may not be directly related to the role of PHT4;1 in SA regulation. It is also possible that there are additional factor(s) affecting circadian expression of the *PHT4;1* gene and/or phosphate transport activity of the PHT4;1 protein. Alternatively, CCA1 and its close homolog LHY could regulate expression of multiple defense genes, including both positive and negative SA regulators. Thus in the CCA1 and LHY loss of function background, the effect on SA accumulation could be negated by the changes of these two opposing groups of SA genes. Additional biochemical, genetic, and molecular studies are required to further elucidate the biological relevance of CCA1 binding on the *PHT4;1* promoter in terms of phosphate transport and defense regulation.

## Conflict of Interest Statement

The authors declare that the research was conducted in the absence of any commercial or financial relationships that could be construed as a potential conflict of interest.
